# Awareness of Genitourinary Cancers Risk Factors—A 2024 Population-Based Cross-Sectional Study in Poland

**DOI:** 10.3389/ijph.2024.1607264

**Published:** 2024-06-21

**Authors:** Gabriela Moczeniat, Mateusz Jankowski, Aneta Duda-Zalewska, Mariusz Gujski

**Affiliations:** ^1^ Department of Public Health, Medical University of Warsaw, Warsaw, Poland; ^2^ Department of Urology, Mazovia Hospital Warsaw, Warsaw, Poland; ^3^ School of Public Health, Centre of Postgraduate Medical Education, Warsaw, Poland

**Keywords:** genitourinary diseases, cancer, preventive health services, public awareness, risk factors

## Abstract

**Objective:**

This study aimed to assess the awareness of genitourinary cancers risk factors among adults in Poland and to identify factors associated with public awareness of risk factors for genitourinary cancers.

**Methods:**

This cross-sectional survey was carried out between 1 and 4 March 2024 in a nationwide sample of 2,165 adults in Poland. Quota sampling was used. Data were collected using computer-assisted web interview (CAWI) method.

**Results:**

Regardless of the type of cancer (kidney, bladder, or prostate cancer), a family history of cancer was the most recognized risk factor indicated by over half of respondents. Over one-third were aware that chemical exposure increases the risk for bladder cancer (39.4%) or prostate cancer (34.2%). Smoking was recognized as a risk factor for kidney cancer by 40.6% of respondents. Female gender, having higher education, being occupationally active and the presence of chronic diseases were the most important factors (*p* < 0.05) associated with a higher level of awareness of genitourinary cancers risk factors.

**Conclusion:**

This study revealed gaps in public awareness of genitourinary cancers risk factors among adults in Poland, especially lifestyle-related and workplace-related risk factors.

## Introduction

Genitourinary cancers include cancers of the urinary system, as well as the reproductive tract [1]. Kidney, bladder, and prostate cancers are the most common genitourinary cancers posing significant health burden [1–3]. Between 1990 and 2019, the global incidence of kidney cancer increased by 155%, bladder cancer by 123%, and prostate cancer by 169% [2]. Population aging will increase the burden of genitourinary cancers [4]. Regular screening is a key preventive measure for early detection of genitourinary cancer [5]. Symptoms differ by type of genitourinary cancer, but most of them include trouble urinating, haematuria, and pain (mostly located in the back, lumps, or abdomen) [6, 7]. However, prostate cancer is often asymptomatic making early detection difficult [7].

Public awareness of genitourinary cancer risk factors is necessary to assess individual health risks and implement regular screening. Smoking is the most important lifestyle-related risk factor for genitourinary cancer [8]. Obesity and some types of diet may also increase the risk of genitourinary cancer [9]. Smoking prevalence in Poland is estimated at 28.8% of the adult population [10] and the prevalence of overweight or obesity is 42.2% and 16.4% respectively [11], so lifestyle-related risk factors significantly contribute to the genitourinary cancer burden in Poland. Chemical exposures, especially environmental and occupational exposures are also well-documented risk factors for genitourinary cancers [12]. Patients with arterial hypertension are also at higher risk of some types of genitourinary cancers, like kidney cancer [13]. Scientific data confirmed the link between chronic use of analgesics, history of chemotherapy or radiation therapy as well as long-term dialysis treatment, and increased risk for genitourinary cancers [14–16]. Genetic predisposition (family history of genitourinary cancers), male gender, and older age also increase the risk for genitourinary cancers [17].

There is a high burden of genitourinary cancers in Poland [18, 19]. Prostate cancer is the most common cancer in men, accounting for 20.6% of all cancer cases in men [18]. Bladder cancer is the fourth most common cancer in men [18]. Kidney cancer accounts for 5% of cancer in men and 3% in women [19]. Moreover, in Poland, the prevalence of genitourinary cancer risk factors, such as smoking [10] and obesity [20] is high. Population aging observed in Poland may also contribute to the growing genitourinary cancer burden. In Poland, educational campaigns on genitourinary cancer are mostly limited to those focused on prostate cancer during the “Movember” annual event, supported by national public health institutions [21]. Moreover, education on different types of cancers (including kidney and bladder cancer) is carried out by non-governmental organizations (NGOs), including those run by patient representatives [22]. Physicians, including general practitioners, are encouraged to provide basic education on cancer risk factors as a part of preventive services, but the involvement of physicians in health education is limited [23]. Nationwide data on public awareness of genitourinary cancer risk factors may inform policymakers and healthcare professionals on current needs for the prevention and control of genitourinary cancers [22, 23].

This study aimed to assess the awareness of genitourinary cancers risk factors among adults in Poland as well as to identify factors associated with public awareness of risk factors for genitourinary cancers.

## Methods

### Study Design and Population

This population-based cross-sectional study was carried out in Poland, between 1 and 4 March 2024 on a nationwide sample of 2,165 adults in Poland. Data were collected using the computer-assisted web interview (CAWI) technique. A professional public opinion research agency (Nationwide Research Panel Ariadna in Warsaw) was contracted to collect data on behalf of the authors [24]. An online panel of more than 100,000 verified persons managed by the research agency was used to recruit participants to form a nationwide sample of the Polish adult population concerning demographic characteristics (gender, age, and place of residence). The population was first divided into subgroups based on mutual exclusivity. Then, respondents were chosen using a stratification model and quota sampling. Demographic data on the age, gender, and place of residence from the Population Yearbook of the Central Statistical Office of the Republic of Poland [25], were used in the stratification model. Every participant received a unique URL to the research website via e-mail that granted a single access to the survey. A text message reminder was also sent. Participation in the study was voluntary and informed consent was collected from all participants. If the selected respondent refused to participate in the survey, the next participant from the list was invited in line with the demographic criteria. The overall response rate was estimated at 22%.

The same methodology was used in previous population-based cross-sectional studies in Poland [26, 27].

This study was approved on 05 February 2024, by the Ethical Board at the Medical University of Warsaw, document signature AKBE/43/2024. All procedures followed the followed the Declaration of Helsinki.

### Study Questionnaire

The study questionnaire was based on a review of the literature on public awareness of genitourinary cancers and their risk factors, including data published by the American Cancer Society [28, 29]. The study questionnaire included 8 questions (5 multiple-choice, 3 single-choice) on genitourinary cancer risk factors and prevention methods. Moreover, a set of questions on the health status of respondents, and sociodemographic characteristics was addressed. A pilot study was carried out, and 8 adults filled the questionnaire twice, 7 days apart. After the pilot study, one question and three answers in multiple-choice questions were revised to improve the clarity of the text.

Respondents were asked about risk factors for genitourinary cancers using the following multiple-choice questions: “What do you think are the risk factors for (1) kidney cancer; (2) bladder cancer; (3) prostate cancer?” With 6–8 answers. Each risk factor was required to answer yes or no. As the study aim was the assessment of risk factors for a group of diseases (genitourinary cancers), questions were focused directly on this group of cancers rather than general cancer knowledge. A similar type of question was used in the study by Luryi et al. in the study on public awareness of head and neck cancers and their risk factors [30]. The list of risk factors for genitourinary cancers included in the multi-choice questions was based on the most common genitourinary cancer risk factors listed by the American Cancer Society [28].

The presence of chronic diseases was self-reported and based on the following question “Do you have chronic diseases or long-term health problems lasting at least 6 months? (yes or no).”

Questions on sociodemographic characteristics addressed: gender, age, marital status, having higher education (yes/no), having children (yes/no), place of residence, and occupational activity (active—currently employed or self-employed; passive—unemployed, retired, or student).

### Statistics

Statistical testing was completed using procedures available in SPSS v.29 (IBM, Armonk, NY, United States). The distribution of categorical variables was presented with frequencies and proportions. Cross-tabulations and chi-square tests were used for the comparison of categorical variables. Multivariable logistic regression analyses were used to identify factors associated with public awareness of genitourinary cancers risk factors. Five best-documented risk factors for genitourinary cancers—(1) smoking, (2) family history of genitourinary cancers, (3) male gender, (4) obesity; and (5) chemical exposures were considered separately as dependent variables in the model. For multivariable logistic regression, it was assumed that if the respondent indicated a given factor as a risk factor for any of the analyzed genitourinary cancers (kidney, bladder, or prostate cancer), the respondent was aware that the given factor increased the risk of genitourinary cancer. The strength of the association was measured by the odds ratio (OR) and 95% confidence intervals (CI). Statistical significance was based on the criterion *p* < 0.05.

## Results

The mean age of respondents was 49.2 ± 16.2 years, 53% of respondents were females, and 41.7% had chronic diseases (Table 1).

**TABLE 1:** Characteristics of the study population, 2024 (*n* = 2,165) (Warsaw, Poland, 2024).

Variables	Total *n* = 2,165
	*n* (%)
Gender	
female	1,148 (53.0)
male	1,017 (47.0)
Age [years]	
18–34	488 (22.5)
35–49	600 (27.7)
50–64	604 (27.9)
65+	473 (21.8)
Marital status	
single	507 (23.4)
married	1,174 (54.2)
informal relationship	308 (14.2)
divorced or widowed	176 (8.1)
Educational level	
higher	985 (45.5)
less than higher	1,180 (54.5)
Having children	
yes	1,496 (69.1)
no	669 (30.9)
Place of residence	
rural	859 (39.7)
city below 20,000 residents	258 (11.9)
city between 20,000–99,999 residents	435 (20.1)
city between 100,000–499,999 residents	357 (16.5)
city above 500,000 residents	256 (11.8)
Occupational activity	
employed/self-employed	1,209 (55.8)
passive (unemployed or retired)	956 (44.2)
Presence of chronic diseases	
yes	902 (41.7)
no	1,263 (58.3)

### Public Awareness of Genitourinary Cancers Risk Factors

Public awareness of genitourinary cancers risk factors is presented in Table 2. Regardless of the type of cancer (kidney, bladder, or prostate cancer), a family history of cancer was the most recognized risk factor indicated by over half of respondents. Over half of the respondents were aware that chemical exposure increases the risk for kidney cancer (50.1%). However, approximately one-third were aware that chemical exposure increases the risk for bladder cancer (39.4%) or prostate cancer (34.2%). Smoking was recognized as a risk factor for kidney cancer by 40.6% of respondents and only 35.3% of respondents indicated smoking as a risk factor for bladder cancer. Less than 30% of respondents were aware that chronic use of analgesics or long-term dialysis treatment increases the risk for kidney cancer (Table 2). Over half of the respondents (51.9%) were aware that chronic bladder irritation and infections increase the risk for bladder cancer. Older age was recognized as a risk factor for prostate cancer by 53.4% of respondents.

**TABLE 2:** Public awareness of genitourinary cancers risk factors, Poland, 2024 (*n* = 2,165) (Warsaw, Poland, 2024).

Variable	Overall (*n* = 2,165)	
	n	%
What do you think are the risk factors for kidney cancer? Multiple-choice question; positive answers)		
smoking	878	40.6
obesity	712	32.9
male gender	182	8.4
arterial hypertension	495	22.9
chemical exposures like workplace exposure to certain substances, such as heavy metals, tannins, asbestos	1,084	50.1
family history of kidney cancer	1,275	58.9
chronic use of analgesic	634	29.3
long-term dialysis treatment	618	28.5
What do you think are the risk factors for bladder cancer? (Multiple-choice question; positive answers)		
smoking	764	35.3
chemical exposures like working in rubber, leather, textiles, aluminum industries	853	39.4
older age	671	31.0
male gender	298	13.8
family history of bladder cancer	1,161	53.6
arsenic in drinking water	723	33.4
chronic bladder irritation and infections	1,124	51.9
history of chemotherapy or radiation therapy	422	19.5
What do you think are the risk factors for prostate cancer? Multiple-choice question; positive answers)		
older age	1,157	53.4
family history of prostate cancer	1,275	58.9
smoking	823	38.0
obesity	542	25.0
diet (high consumption of meat and dairy products)	406	18.8
chemical exposures like pesticides or cadmium	740	34.2

### Sociodemographic Differences in Public Awareness of Genitourinary Cancers Risk Factors

There were sociodemographic differences in public awareness of risk factors for kidney cancer (Table 3), bladder cancer (Table 4), and prostate cancer (Table 5). In general, respondents with chronic diseases as well as those with higher education more often correctly indicated risk factors for genitourinary cancers (*p* < 0.05). Females more often correctly indicated risk factors for kidney cancer when compared to males (Table 3). There were gender differences in the percentage of patients who correctly indicated risk factors for bladder cancer (Table 4). Moreover, awareness of risk factors for prostate cancer differed by age (Table 5).

**TABLE 3:** Awareness of risk factors for kidney cancer by sociodemographic factors (*n* = 2,165) (Warsaw, Poland, 2024).

Variables	Risk factors for kidney cancer - percentage of respondents who answered “yes” by sociodemographic factors															
	Smoking		Obesity		Male gender		Arterial hypertension		Chemical exposures		Family history of kidney cancer		Chronic use of analgesic		Long-term dialysis treatment	
	n (%)	*p*	n (%)	*p*	n (%)	*p*	n (%)	*p*	n (%)	*p*	n (%)	*p*	n (%)	*p*	n (%)	*p*
Gender																
female	449 (39.1)	0.1	367 (32.0)	0.3	84 (7.3)	0.05	275 (24.0)	0.2	632 (55.1)	<0.001	751 (65.4)	<0.001	359 (31.3)	0.03	391 (34.1)	<0.001
male	429 (42.2)		345 (33.9)		98 (9.6)		220 (21.6)		452 (44.4)		524 (51.5)		275 (27.0)		227 (22.3)	
Age [years]																
18–34	212 (43.4)	0.2	182 (37.3)	0.05	59 (12.1)	0.01	131 (26.8)	0.03	235 (48.2)	0.1	263 (53.9)	0.04	158 (32.4)	0.3	133 (27.3)	0.03
35–49	254 (42.3)		203 (33.8)		48 (8.0)		118 (19.7)		285 (47.5)		350 (58.3)		180 (30.0)		151 (25.2)	
50–64	229 (37.9)		184 (30.5)		45 (7.5)		144 (23.8)		307 (50.8)		366 (60.6)		166 (27.5)		197 (32.6)	
65+	183 (38.7)		143 (30.2)		30 (6.3)		102 (21.6)		257 (54.3)		296 (62.6)		130 (27.5)		137 (29.0)	
Marital status																
single	210 (41.4)	0.4	187 (36.9)	0.1	38 (7.5)	0.04	122 (24.1)	0.5	246 (48.5)	0.4	286 (56.4)	0.5	140 (27.6)	0.01	131 (25.8)	0.5
married	481 (41.0)		369 (31.4)		111 (9.5)		271 (23.1)		603 (51.4)		704 (60.0)		335 (28.5)		345 (29.4)	
informal relationship	126 (40.9)		105 (34.1)		27 (8.8)		69 (22.4)		155 (50.3)		178 (57.8)		114 (37.0)		92 (29.9)	
divorced or widowed	61 (34.7)		51 (29.0)		6 (3.4)		33 (18.8)		80 (45.5)		107 (60.8)		45 (25.6)		50 (28.4)	
Educational level																
higher	440 (44.7)	<0.001	368 (37.4)	<0.001	94 (9.5)	0.08	264 (26.8)	<0.001	562 (57.1)	<0.001	640 (65.0)	<0.001	325 (33.0)	<0.001	281 (28.5)	0.9
less than higher	438 (37.1)		344 (29.2)		88 (7.5)		231 (19.6)		522 (44.2)		635 (53.8)		309 (26.2)		337 (28.6)	
Having children																
yes	587 (39.2)	0.1	472 (31.6)	0.04	120 (8.0)	0.3	340 (22.7)	0.8	763 (51.0)	0.2	906 (60.6)	0.02	430 (28.7)	0.4	444 (29.7)	0.1
no	291 (43.5)		240 (35.9)		62 (9.3)		155 (23.2)		321 (48.0)		369 (55.2)		204 (30.5)		174 (26.0)	
Place of residence																
rural	337 (39.2)	0.8	256 (29.8)	0.02	65 (7.6)	0.2	185 (21.5)	0.04	408 (47.5)	0.1	479 (55.8)	0.06	240 (27.9)	0.7	242 (28.2)	0.8
city below 20,000 residents	105 (40.7)		79 (30.6)		17 (6.6)		45 (17.4)		129 (50.0)		160 (62.0)		73 (28.3)		74 (28.7)	
city between 20,000–99,999 res	176 (40.5)		148 (34.0)		37 (8.5)		105 (24.1)		217 (49.9)		250 (57.5)		129 (29.7)		119 (27.4)	
city between 100,000–499,999 residents	151 (42.3)		127 (35.6)		33 (9.2)		88 (24.6)		184 (51.5)		223 (62.5)		112 (31.4)		111 (31.1)	
city above 500,000 residents	109 (42.6)		102 (39.8)		30 (11.7)		72 (28.1)		146 (57.0)		163 (63.7)		80 (31.3)		72 (28.1)	
Occupational activity																
employed/self-employed	498 (41.2)	0.5	427 (35.3)	0.01	121 (10.0)	0.003	283 (23.4)	0.5	609 (50.4)	0.8	695 (57.5)	0.1	366 (30.3)	0.3	349 (28.9)	0.7
passive	380 (39.7)		285 (29.8)		61 (6.4)		212 (22.2)		475 (49.7)		580 (60.7)		268 (28.0)		269 (28.1)	
Presence of chronic diseases																
yes	378 (41.9)	0.3	333 (36.9)	<0.001	93 (10.3)	0.01	243 (26.9)	<0.001	482 (53.4)	0.01	592 (65.6)	<0.001	295 (32.7)	0.003	286 (31.7)	0.01
no	500 (39.6)		379 (30.0)		89 (7.0)		252 (20.0)		602 (47.7)		683 (54.1)		339 (26.8)		332 (26.3)	

**TABLE 4:** Awareness of risk factors for bladder cancer by sociodemographic factors (*n* = 2,165) (Warsaw, Poland, 2024).

Variables	Risk factors for bladder cancer—percentage of respondents who answered “yes” by sociodemographic factors																
	Smoking		Chemical exposures		Older age		Male gender		Family history of bladder cancer		arsenic in drinking water			chronic bladder irritation		chemotherapy or radiation therapy	
	n (%)	*p*	n (%)	*p*	n (%)	*p*	n (%)	*p*	n (%)	*p*	n (%)	*p*	n (%)		*p*	n (%)	*p*
Gender																	
female	392 (34.1)	0.2	482 (42.0)	0.01	319 (27.8)	<0.001	138 (12.0)	0.01	689 (60.0)	<0.001	417 (36.3)	0.002	656 (57.1)		<0.001	248 (21.6)	0.01
male	372 (36.6)		371 (36.5)		352 (34.6)		160 (15.7)		472 (46.4)		306 (30.1)		468 (46.0)			174 (17.1)	
Age [years]																	
18–34	172 (35.2)	0.8	175 (35.9)	0.01	153 (31.4)	<0.001	81 (16.6)	0.09	253 (51.8)	0.04	178 (36.5)	0.1	246 (50.4)		<0.001	106 (21.7)	0.04
35–49	222 (37.0)		213 (35.5)		166 (27.7)		83 (13.8)		298 (49.7)		180 (30.0)		281 (46.8)			128 (21.3)	
50–64	208 (34.4)		261 (43.2)		171 (28.3)		68 (11.3)		342 (56.6)		201 (33.3)		314 (52.0)			115 (19.0)	
65+	162 (34.2)		204 (43.1)		181 (38.3)		66 (14.0)		268 (56.7)		164 (34.7)		283 (59.8)			73 (15.4)	
Marital status																	
single	173 (34.1)	0.7	191 (37.7)	0.5	162 (32.0)	0.4	69 (13.6)	0.4	269 (53.1)	0.5	164 (32.3)	0.5	239 (47.1)		0.1	97 (19.1)	0.6
married	426 (36.3)		475 (40.5)		361 (30.7)		167 (14.2)		637 (54.3)		386 (32.9)		627 (53.4)			230 (19.6)	
informal relationship	108 (35.1)		114 (37.0)		102 (33.1)		45 (14.6)		170 (55.2)		114 (37.0)		162 (52.6)			66 (21.4)	
divorced or widowed	57 (32.4)		73 (41.5)		46 (26.1)		17 (9.7)		85 (48.3)		59 (33.5)		96 (54.5)			29 (16.5)	
Educational level																	
higher	393 (39.9)	<0.001	442 (44.9)	<0.001	328 (33.3)	0.03	151 (15.3)	0.05	568 (57.7)	<0.001	378 (38.4)	<0.001	547 (55.5)		0.002	214 (21.7)	0.02
less than higher	371 (31.4)		411 (34.8)		343 (29.1)		147 (12.5)		593 (50.3)		345 (29.2)		577 (48.9)			208 (17.6)	
Having children																	
yes	518 (34.6)	0.3	609 (40.7)	0.06	446 (29.8)	0.1	194 (13.0)	0.1	829 (55.4)	0.01	502 (33.6)	0.8	808 (54.0)		0.004	290 (19.4)	0.9
no	246 (36.8)		244 (36.5)		225 (33.6)		104 (15.5)		332 (49.6)		221 (33.0)		316 (47.2)			132 (19.7)	
Place of residence																	
rural	294 (34.2)	0.6	314 (36.6)	0.2	246 (28.6)	0.4	129 (15.0)	0.4	438 (51.0)	0.07	273 (31.8)	0.2	406 (47.3)		0.002	160 (18.6)	0.8
city below 20,000 residents	84 (32.6)		107 (41.5)		84 (32.6)		35 (13.6)		142 (55.0)		76 (29.5)		140 (54.3)			60 (19.4)	
city between 20,000–99,999 res	154 (35.4)		187 (43.0)		143 (32.9)		48 (11.0)		224 (51.5)		155 (35.6)		225 (51.7)			94 (21.6)	
city between 100,000–499,999 residents	136 (38.1)		137 (38.4)		116 (32.5)		50 (14.0)		207 (58.0)		130 (36.4)		211 (59.1)			71 (19.9)	
city above 500,000 residents	96 (37.5)		108 (42.2)		82 (32.0)		36 (14.1)		150 (58.6)		89 (34.8)		142 (55.5)			47 (18.4)	
Occupational activity																	
employed/self-employed	424 (35.1)	0.8	477 (39.5)	0.9	373 (30.9)	0.9	179 (14.8)	0.1	637 (52.7)	0.3	404 (33.4)	0.9	593 (49.0)		0.003	260 (21.5)	0.01
passive	340 (35.6)		376 (39.3)		298 (31.2)		119 (12.4)		524 (54.8)		319 (33.4)		531 (55.5)			162 (16.9)	
Presence of chronic diseases																	
yes	352 (39.0)	0.002	398 (44.1)	<0.001	316 (35.0)	<0.001	151 (16.7)	<0.001	537 (59.5)	<0.001	335 (37.1)	0.002	521 (57.8)		<0.001	194 (21.5)	0.04
no	412 (32.6)		455 (36.0)		355 (28.1)		147 (11.6)		624 (49.4)		388 (30.7)		603 (47.7)			228 (18.1)	

**TABLE 5:** Awareness of risk factors for prostate cancer by sociodemographic factors (n = 2,165) (Warsaw, Poland, 2024).

Variables	Risk factors for prostate cancer - percentage of respondents who answered “yes” by sociodemographic factors											
	Older age		Family history of prostate cancer		Smoking		Obesity		Diet		Chemical exposures	
	n (%)	*p*	n (%)	*p*	n (%)	*p*	n (%)	*p*	n (%)	*p*	n (%)	*p*
Gender												
female	623 (54.3)	0.4	745 (64.9)	<0.001	445 (38.8)	0.4	267 (23.3)	0.04	231 (20.1)	0.08	402 (35.0)	0.4
male	534 (52.5)		530 (52.1)		378 (37.2)		275 (27.0)		175 (17.2)		338 (33.2)	
Age [years]												
18–34	226 (46.3)	<0.001	259 (53.1)	0.02	203 (41.6)	0.001	140 (28.7)	0.02	110 (22.5)	0.04	175 (35.9)	0.8
35–49	299 (49.8)		355 (59.2)		255 (42.5)		156 (26.0)		111 (18.5)		205 (34.2)	
50–64	332 (55.0)		374 (61.9)		210 (34.8)		152 (25.2)		112 (18.5)		201 (33.3)	
65+	300 (63.4)		287 (60.7)		155 (32.8)		94 (19.9)		73 (15.4)		159 (33.6)	
Marital status												
single	246 (48.5)	0.1	275 (54.2)	0.01	197 (38.9)	0.4	131 (25.8)	0.06	92 (18.1)	0.01	169 (33.3)	0.7
married	650 (55.4)		722 (61.5)		449 (38.2)		311 (26.5)		228 (19.4)		412 (35.1)	
informal relationship	167 (54.2)		185 (60.1)		120 (39.0)		68 (22.1)		68 (22.1)		105 (34.1)	
divorced or widowed	94 (53.4)		93 (52.8)		57 (32.4)		32 (18.2)		18 (10.2)		54 (30.7)	
Educational level												
higher	564 (57.3)	0.001	630 (64.0)	<0.001	424 (43.0)	<0.001	287 (29.1)	<0.001	246 (25.0)	<0.001	397 (40.3)	<0.001
less than higher	593 (50.3)		645 (54.7)		399 (33.8)		255 (21.6)		160 (13.6)		343 (29.1)	
Having children												
yes	815 (54.5)	0.1	906 (60.6)	0.02	549 (36.7)	0.06	363 (24.3)	0.2	262 (17.5)	0.03	518 (34.6)	0.5
no	342 (51.1)		369 (55.2)		274 (41.0)		179 (26.8)		144 (21.5)		222 (33.2)	
Place of residence												
rural	438 (51.0)	0.1	479 (55.8)	0.01	345 (40.2)	0.2	204 (23.7)	0.8	146 (17.0)	0.07	300 (34.9)	0.5
city below 20,000 residents	147 (57.0)		145 (56.2)		95 (36.8)		70 (27.1)		41 (15.9)		88 (34.1)	
city between 20,000–99,999 residents	222 (51.0)		255 (58.6)		146 (33.6)		109 (25.1)		82 (18.9)		134 (30.8)	
city between 100,000–499,999 res	202 (56.6)		233 (65.3)		139 (38.9)		91 (25.5)		77 (21.6)		124 (34.7)	
city above 500,000 residents	148 (57.8)		163 (63.7)		98 (38.3)		68 (26.6)		60 (23.4)		94 (36.7)	
Occupational activity												
employed/self-employed	626 (51.8)	0.1	707 (58.5)	0.7	490 (40.5)	0.01	331 (27.4)	0.01	251 (20.8)	0.01	423 (35.0)	0.4
passive	531 (55.5)		568 (59.4)		333 (34.8)		211 (22.1)		155 (16.2)		317 (33.2)	
Presence of chronic diseases												
yes	542 (60.1)	<0.001	596 (66.1)	<0.001	366 (40.6)	0.04	261 (28.9)	<0.001	196 (21.7)	0.003	346 (38.4)	<0.001
no	615 (48.7)		679 (53.8)		457 (36.2)		281 (22.2)		210 (16.6)		394 (31.2)	

### Factors Associated With Public Awareness of Genitourinary Cancers Risk Factors

In multivariable logistic regression, awareness of 5 different risk factors for genitourinary cancers was analyzed (Table 6). Age 18–49 (*p* < 0.05), having higher education (*p* < 0.001), having children (*p* < 0.001) as well as the presence of chronic diseases (*p* = 0.02) were associated with higher awareness of smoking as a risk factor for genitourinary cancers. Female gender (*p* < 0.001), marital status (*p* < 0.05), having higher education (*p* = 0.03), and the presence of chronic diseases (*p* < 0.001) were associated with higher awareness of family history of genitourinary cancers as a risk factor for genitourinary cancers. Male gender (*p* = 0.04), being employed or self-employed (*p* = 0.03), and the presence of chronic diseases (*p* < 0.001) were associated with higher awareness of male gender as a risk factor for genitourinary cancers. Age 18–34 years (*p* < 0.003), having higher education (*p* = 0.002), being employed or self-employed (*p* = 0.02), and the presence of chronic diseases (*p* < 0.001) were associated with higher awareness of obesity as a risk factor for genitourinary cancers. Female gender (*p* = 0.001), having higher education (*p* < 0.001), being employed or self-employed (*p* = 0.04), and the presence of chronic diseases (*p* = 0.01) were associated with higher awareness of chemical exposures as a risk factor for genitourinary cancers (Table 6).

**TABLE 6:** Factors associated with awareness of genitourinary cancers risk factors (*n* = 2,165) (Warsaw, Poland, 2024).

Variables	Factors associated with awareness of risk factors for genitourinary cancers (kidney, bladder or prostate cancer)									
	Smoking		Family history of genitourinary cancer		Male gender		Obesity		Chemical exposures	
	OR (95%CI)	*p*	OR (95%CI)	*p*	OR (95%CI)	*p*	OR (95%CI)	*p*	OR (95%CI)	*p*
Gender										
female	0.92 (0.77–1.09)	0.3	1.90 (1.56–2.33)	<0.001	Reference		0.91 (0.76–1.09)	0.3	1.35 (1.13–1.62)	0.001
male	Reference		Reference		1.28 (1.02–1.61)	0.04	Reference		Reference	
Age [years]										
18–34	1.57 (1.12–2.19)	0.01	0.70 (0.47–1.02)	0.06	1.49 (0.96–2.30)	0.8	1.69 (1.20–2.38)	0.003	0.93 (0.66–1.31)	0.7
35–49	1.38 (1.02–1.86)	0.04	0.79 (0.56–1.13)	0.2	0.95 (0.63–1.43)	0.8	1.26 (0.92–1.73)	0.1	0.76 (0.55–1.03)	0.1
50–64	0.95 (0.73–1.25)	0.7	0.85 (0.62–1.17)	0.3	0.77 (0.53–1.13)	0.2	1.08 (0.81–1.43)	0.6	0.94 (0.71–1.25)	0.7
65+	Reference		Reference		Reference		Reference		Reference	
Marital status										
single	Reference		1.69 (1.10–2.61)	0.02	1.16 (0.65–2.04)	0.6	1.32 (0.89–1.97)	0.2	1.09 (0.74–1.61)	0.7
married	1.21 (0.93–1.58)	0.2	1.66 (1.15–2.39)	0.01	1.55 (0.93–2.56)	0.1	1.17 (0.82–1.65)	0.4	1.29 (0.92–1.81)	0.1
informal relationship	1.05 (0.78–1.41)	0.7	1.77 (1.13–2.77)	0.01	1.23 (0.69–2.22)	0.5	1.09 (0.72–1.65)	0.7	1.15 (0.77–1.73)	0.5
divorced or widowed	1.00 (0.68–1.46)	0.9	Reference		Reference		Reference		Reference	
Educational level										
higher	1.36 (1.14–1.62)	<0.001	1.58 (1.29–1.94)	<0.001	1.08 (0.86–1.37)	0.5	1.32 (1.10–1.58)	0.002	1.56 (1.30–1.88)	<0.001
less than higher	Reference		Reference		Reference		Reference		Reference	
Having children										
yes	1.36 (1.14–1.62)	<0.001	1.10 (0.82–1.46)	0.5	0.89 (0.64–1.24)	0.5	1.07 (0.83–1.39)	0.6	1.07 (0.83–1.39)	0.6
no	Reference		Reference		Reference		Reference		Reference	
Place of residence										
rural	Reference		Reference		Reference		Reference		Reference	
city below 20,000 residents	0.96 (0.74–1.30)	0.9	1.16 (0.84–1.60)	0.4	0.87 (0.59–1.29)	0.5	1.30 (0.97–1.73)	0.1	1.19 (0.89–1.60)	0.2
city between 20,000–99,999 residents	0.88 (0.70–1.12)	0.3	1.00 (0.77–1.31)	0.9	0.84 (0.61–1.15)	0.3	1.21 (0.95–1.54)	0.1	1.18 (0.93–1.51)	0.2
city between 100,000–499,999 residents	0.98 (0.76–1.26)	0.9	1.25 (0.93–1.68)	0.1	1.06 (0.76–1.46)	0.7	1.20 (0.93–1.55)	0.2	0.99 (0.77–1.29)	0.9
city above 500,000 residents	0.87 (0.66–1.17)	0.4	1.35 (0.96–1.91)	0.1	1.07 (0.74–1.54)	0.7	1.31 (0.98–1.75)	0.1	1.13 (0.84–1.52)	0.4
Occupational activity										
employed/self-employed	1.13 (0.91–1.40)	0.3	1.22 (0.96–1.55)	0.1	1.40 (1.04–1.88)	0.03	1.30 (1.04–1.62)	0.02	1.25 (1.01–1.55)	0.04
passive	Reference		Reference		Reference		Reference		Reference	
Presence of chronic diseases										
yes	1.25 (1.04–1.50)	0.02	1.71 (1.38–2.12)	<0.001	1.67 (1.32–2.13)	<0.001	1.44 (1.19–1.74)	<0.001	1.32 (1.09–1.59)	0.01
no	Reference		Reference		Reference		Reference		Reference	

## Discussion

This population-based cross-sectional study provided data on public awareness of genitourinary (kidney, bladder, and prostate) cancers risk factors among adults in Poland. There was relatively low public awareness of genitourinary cancers risk factors, especially lifestyle-related and workplace-related risk factors. There were significant sociodemographic differences in awareness of particular risk factors for genitourinary cancer. Female gender, having higher education, being occupationally active and the presence of chronic diseases were the most important factors associated with a higher level of awareness of genitourinary cancers risk factors.

Awareness of genitourinary cancers risk factors may impact individuals’ behaviors related to cancer screening and prevention, including the prevalence of testing as well as lifestyle changes [8, 9]. It is estimated that one-quarter of adults in Poland are smokers [10]. Smoking increases the risk for most of the genitourinary cancers [8]. In this study, over 60% of adults in Poland were not aware of the link between smoking and kidney, bladder, or prostate cancer. This finding is in line with previously published data on insufficient counseling and knowledge of genitourinary cancers as tobacco-related diseases [31]. Urologists may play an important role in tobacco counseling, as patients who receive smoking cessation advice from their urologist are over 2 times more likely to attempt to quit [32].

Obesity is a significant risk factor for numerous cancers, including genitourinary cancers [9]. In this study majority (approximately 70%) of respondents were not aware of obesity as a risk factor for genitourinary cancers. Education on obesity-related cancers is difficult due to the reality of obesity stigma [33]. Nevertheless, there is a need to educate people on health consequences of obesity, especially in countries with high overweight and obesity prevalence, like Poland [23].

Chemical exposures, especially workplace-related exposures to heavy metals, tannins, and asbestos, working in agriculture where pesticides are used, or working in rubber, leather, textiles, or aluminum industries may increase risks of genitourinary cancers [12]. Workplace-related health risks are controlled within occupational medicine screening and surveillance [34]. However, public awareness of chemical exposures that increase cancer risks may motivate workers to comply with rules on security measures in the workplace or to change industries [12, 34]. Occupational medicine professionals may be involved in workers’ education on genitourinary cancers.

Family history of cancers is important data that influences the frequency of cancer screening [35]. Out of all genitourinary cancers risk factors analyzed in this study, a family history of cancer was the most recognized risk factor indicated by over half of respondents. Patients with a family history of genitourinary cancers should be educated on the importance of cancer screening [36].

Genitourinary cancers are more prevalent among men [2]. However, in this study, approximately 10% of respondents were aware of gender differences in the prevalence and risk of genitourinary cancers. Older age was correctly indicated as a risk factor for prostate cancer by over half of respondents, whereas only one-third of respondents indicated older age as a risk factor for bladder cancer. Education on genitourinary cancers risk factors should include activities targeted to older adults, especially older males, who are at higher risk for genitourinary diseases [17].

In this study, less than 30% of respondents were aware of genitourinary cancer risk factors related to healthcare procedures and treatment methods, including analgesics use, long-term dialysis treatment, and chemotherapy or radiation therapy [14–16]. Clinical guidelines should be regularly revised and updated to reduce the risk of genitourinary cancers related to healthcare procedures [14–16].

Sociodemographic differences in public awareness of different risk factors for genitourinary cancers indicated health inequalities in the knowledge of cancers in Poland. This finding suggests the need to prepare tailored activities, including those addressed to local communities [37]. Out of 8 different factors analyzed in this study, the presence of chronic diseases was associated with awareness of all 5 risk factors analyzed in multivariable logistic regression analyses. Health status and personal experience related to visits to healthcare facilities, as well as health education by physicians during these visits, may shape individuals’ knowledge of cancer [38]. Education is a well-reported factor associated with knowledge of health and health literacy levels [39]. In this study, having higher education was significantly associated with higher awareness of risk factors for genitourinary cancers. Occupationally active respondents were also more aware of genitourinary disease risk factors, which may result from the fact that they are covered by occupational medicine services and health education in the workplace [40]. Female gender was associated with higher awareness of gender and chemical exposures as risk factors for genitourinary cancers, whereas male gender was associated with higher awareness of gender differences in the risk of genitourinary diseases. There is a need for further education on genitourinary cancers targeted to males.

This study revealed gaps in public awareness of genitourinary cancers risk factors among adults in Poland. Educational campaigns on genitourinary cancers risk factors (especially those lifestyle-related) should be implemented. Public interventions on genitourinary cancers should be targeted to populations with the lowest awareness of genitourinary cancers, including those without higher education, males, and pensioners. Improvement of public knowledge on genitourinary cancers should be one of the targets of cancer policy in Poland. Findings from this study may pose a basis for international comparisons, especially between European countries.

The study questionnaire was limited to the most common risk factors for genitourinary diseases. There was no direct face-to-face interaction with the respondents, so there was no possibility to judge the respondents’ abilities and capacity to comprehend the questions posed. The cross-sectional design limiting some casual inferences and the nature of self-reported findings are also limitations of this study. Data on chronic conditions were self-reported and medical records were not verified. This study was carried out in a nationwide sample of adults in Poland and further studies in high-risk populations are needed.

### Conclusion

Findings from this study revealed gaps in public awareness of genitourinary cancers risk factors among adults in Poland. Education on lifestyle-related and workplace-related risk factors for genitourinary cancers should be implemented. Gender, educational level, and health status (presence of chronic diseases) were the most important factors associated with public awareness of genitourinary cancers risk factors. National cancer strategies should pay more attention to education on genitourinary cancers risk factors.

## Data Availability

The dataset used to conduct the analyses is available from corresponding author upon reasonable request.
